# Attitude of mental health care professionals toward borderline personality disorder sufferers in Egypt

**DOI:** 10.1192/j.eurpsy.2022.1719

**Published:** 2022-09-01

**Authors:** A. Abdelkarim, A. Radwan

**Affiliations:** 1 Alexandria Faculty of Medicine, Psychiatry, Alexandria, Egypt; 2 Faculty of medicine - Alexandria university, Psychiatry, Alexandria, Egypt

**Keywords:** Egypt, attitude, borderline personality disorder

## Abstract

**Introduction:**

Awareness and knowledge about borderline personality disorder (BPD) is growing during the last decade in Egypt. Yet little is known about the attitude of mental health care providers toward BPD sufferers. Stigma and judgments among health care providers will affect the quality of services provided to these group of patients. Determining those judgments and pointing to the stigma between health care providers will help improving the quality of care to BPD sufferers.

**Objectives:**

Our objective was to study the attitude of mental health care providers in Egypt toward patients with borderline personality disorder.

**Methods:**

62 mental health care providers, with a majority of psychiatrists, working in Egypt completed the attitude to personality disorder questionnaire “APDQ” designed by Bowers et al. (1998). The questionnaire was disturbed through an online form and knowledge of English was mandatory as it was the language of the questionnaire.

**Results:**

The 62 partcipants of which 74.2% were psychiatrists and 68.7% had more than 5 years experince had a total mean score of APDQ of 138.76. The total mean score of 47 psychiatrists was 137.21 which was significantly lower than the mean score of 15 clinical psychologists and counsellors which scored 146.87.

**Conclusions:**

Whereas mental health care professionals in Egypt had generally positive attitude towards BPD patients, clinical psychologists and counsellors had significantly higher scores in comparison to psychiatrists.

**Disclosure:**

No significant relationships.

